# Ideal cardiovascular health at age 5–6 years and cardiometabolic outcomes in preadolescence

**DOI:** 10.1186/s12966-021-01090-2

**Published:** 2021-03-06

**Authors:** Hester Jaspers Faijer-Westerink, Mette Stavnsbo, Barbara A. Hutten, Mai Chinapaw, Tanja G. M. Vrijkotte

**Affiliations:** 1 Department of Public and Occupational Health, Amsterdam Public Health Research Institute, Amsterdam UMC, University of Amsterdam, PO Box 22700, Meibergdreef 9, room J2-209, 1100 DE Amsterdam, The Netherlands; 2 Department of Sports Science and Physical Education, University of Agder, PO BOX 422, 4604 Kristiansand, Norway; 3 Department of Clinical Epidemiology, Biostatistics and Bioinformatics, Amsterdam Cardiovascular Sciences Research Institute, Amsterdam Public Health Research Institute, Amsterdam UMC, University of Amsterdam, PO Box 22700, Meibergdreef 9, room J1B-209-1, 1100 DE Amsterdam, The Netherlands; 4 Department of Public and Occupational Health, Amsterdam Public Health Research Institute, Amsterdam UMC, Vrije Universiteit, PO Box 7057, van der Boechorststraat 7, 1081 BT, Amsterdam, The Netherlands

**Keywords:** Children, Ideal cardiovascular health, Cardiovascular risk, Epidemiology, Health behaviours

## Abstract

**Background:**

The American Heart Association (AHA) developed a definition of ideal cardiovascular health (ICH) based on the presence of both ideal health behaviours (diet, physical activity, weight status and smoking) and ideal health factors (glucose, total cholesterol and blood pressure levels). However, research of ICH in the paediatric population is scarce. We aimed to study ICH at age 5–6 years by extending the original ICH score with the health behaviours: sleep duration, screen time and prenatal smoke exposure, and to evaluate its association with cardiometabolic outcomes at age 11–12.

**Methods:**

A total of 1666 children aged 5–6 years were selected from the database of the ABCD-study, a prospective cohort study on the health and development of children born in Amsterdam, the Netherlands. Of these, 846 (50.8%) were boys and 1460 (87.6%) had a healthy weight. Data on self-reported health behaviours and health factors were used to calculate the ICH scores (original and extended) by adding the frequency of scoring ‘healthy’ on each indicator, based on international cut-offs. The children were followed up for 6 years and cardiometabolic outcomes (carotid intima-media thickness (CIMT), blood pressure, glucose and lipids) were measured. Associations between ICH (both original and extended) and cardiometabolic outcomes were examined using multivariable regression models.

**Results:**

At age 5–6 years, 11% scored poor (score 1–5), 56% intermediate (score 6–7) and 33% good (score 8–9) on extended ICH. Healthy diet and normal total cholesterol concentrations were the least prevalent. Neither the original nor the extended ICH scores were associated with CIMT at age 11–12. A higher score on the extended ICH was associated with lower total cholesterol (*p* for trend < 0.001), lower systolic (*p* for trend = 0.012) and diastolic blood pressure (*p* for trend = 0.011), and lower body mass index (BMI) (*p* < 0.001) at age 11–12. The original ICH score was associated with lower total cholesterol (p < 0.001) and BMI (*p* < 0.001) only.

**Conclusion:**

Our findings suggest that extending the ICH score in young children with additional health behaviours improves prediction of some cardiometabolic outcomes, but not CIMT in preadolescence, compared to the original ICH score. We would recommend other researchers to incorporate objective measures of health behaviours and longer follow-up to find out whether associations persist into adulthood.

**Supplementary Information:**

The online version contains supplementary material available at 10.1186/s12966-021-01090-2.

## Introduction

Cardiovascular diseases (CVDs) are the leading cause of death and remain one of the most important health challenges worldwide [1]. Although most children are born with optimal cardiovascular health, clustering of CVDs (or cardiometabolic) risk factors already occurs in childhood [2]. As both health behaviours as well as cardiometabolic risk factors track from childhood into adulthood, maintaining cardiovascular health in children is an important step to take [3]. However, to develop successful strategies with special focus on cardiovascular health in children, the most important CVD risk factors in children need to be identified.

To define and measure cardiovascular health of individuals, the American Heart Association (AHA) created the construct of ideal cardiovascular health (ICH). The ICH construct consists of seven metrics: four health behaviours (not smoking, having a healthy weight status, being sufficiently physically active and eating a cardiovascular-healthy diet), and three health factors (healthy levels of total cholesterol, blood pressure and glucose) [4].

.The construct of ICH is already used in population-based studies, especially focusing on adults. Reviews of these studies show that there is an inverse association between ideal ICH metrics and the incidence of CVDs [5–8]. There is also evidence that even one point higher on the ICH metrics, gives a reduction of 19% in cardiovascular related mortality [7]. Prevention strategies focusing on the factors included in the ICH metrics is thus highly recommended. However, studies on the prevalence of ICH in children are scarce [9–12]; only three have been conducted in children > 12 years of age [9, 11, 12]. Further, few studies have investigated the relationship between ICH and cardiometabolic outcomes in populations under the age of 25, but these studies mainly focus on individual components [13–18].

.The definition of ICH in children differs slightly from the adult definition [4]. Smoking is excluded in the ICH definition in children, as children younger than 12 years normally do not smoke. Also, recommended physical activity levels are higher for children: ≥60 min of moderate to vigorous physical activity per day.

Growing evidence shows that the health behaviours included in the ICH construct are not the only risk factors for CVD. In adults, excessive sedentary time and short sleep duration have i.e. shown to be risk factors too. The longitudinal evidence for health effects of sedentary behaviour is inconsistent, but the evidence for screen time alone is more convincing [19]. As health behaviours lead to changes in health factors, adding more components to the ICH construct could make the construct more sensitive for the prediction of cardiometabolic outcomes. Thus we propose an extended version of the ICH definition for the purpose of this study by adding screen time, sleep duration, and prenatal smoke exposure. All three components have been associated with cardiovascular health in children [20, 21].

.The aim of our study was to apply this extended ICH score to a cohort of 5–6 year old children and evaluate its association with cardiometabolic outcomes at age 11–12 years. As children aged 11–12 years old do not yet experience cardiovascular diseases, we focused on the carotid intima-media thickness (CIMT), a surrogate marker for CVD [22], as our primary outcome. We compared the results with the original ICH definition.

## Methods

### Study population and design

This study was part of the Amsterdam Born Children and their Development (ABCD)-study, a prospective cohort study based in the Netherlands. The ABCD-study aims to examine the associations between maternal lifestyle, health and psychosocial circumstances during pregnancy and the child’s health at birth and later in life [23]. Between January 2003 and March 2004, 12,373 pregnant women were approached (> 99% of the target population) during their first prenatal visit to an obstetric care provider, of which 8266 agreed to participate and filled out a pregnancy questionnaire without getting any incentives.

**Fig. 1:**
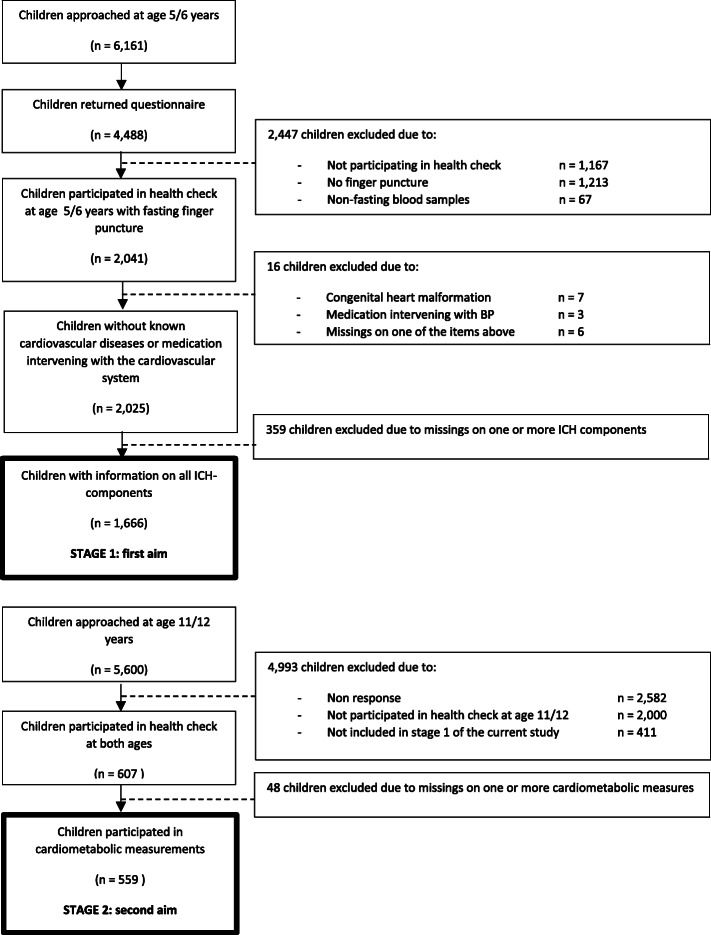
Flowchart of study population, baseline in 2008–2010, follow-up in 2014–2015

Two weeks after their child’s fifth birthday, mothers who gave permission for follow-up and whose addresses could be retrieved (*n* = 6161) received a questionnaire on the health, development and behaviour of their child and an invitation for the child’s health check. The questionnaire was returned for 4488 children, of which 2041 participated in the health check consisting of among other, a finger prick for blood collection after an overnight fast, measurements of height, weight, waist circumference and measurements of blood pressure. After the health check children received a small gift. For those children who participated in the health check, mothers were also asked to fill in a validated food frequency questionnaire (FFQ) [24]. Information on all ICH components was available from 1666 children at age 5–6 years. A randomly selected subgroup of 1081 children were approached for participation in follow-up health measurements at age 11–12 years (March 2015 to June 2016). The health check was similar to the health check at the age of 5, but ultrasound measurements of CIMT were added. For participation children received a gift voucher worth of 10 euros. From this group, 607 children participated at both time points and 559 children had information on the cardiovascular profile. Information from these 559 children were used to investigate the association between ICH at age 5–6 and cardiovascular health at age 11–12. The information of 459 children could be used for the association between ICH at age 5–6 and CIMT. Flowchart of the study population is presented in Fig. 1.

For inclusion in stage 1 of the current study the children had to meet the following criteria: 1) availability of information on maternal smoking status during pregnancy, 2) participating in the 5 year health check with cardiovascular measurements, 3) filled out FFQ, 4) filled out questionnaire on physical activity, sedentary behaviour and sleep duration. For inclusion in stage 2 of the study, children had to participate in the 11 year health check with cardiovascular measurements. Exclusion criteria’s from participating in the study were: non-fasting blood samples, congenital heart malformations or use of medication altering blood pressure or lipid concentrations.

### Demographic information

Ethnicity was based on the mother and her mother’s country of birth, derived from the pregnancy questionnaire and categorized into Dutch, Turkey, Moroccan and Surinamese (the three most common non-Western ethnicities in the Netherlands). The remaining ethnicities were categorized into western and non-western ethnicities. Information on mother’s and father’s weight and height to calculate parent’s body mass index (BMI) was derived from the questionnaire mother’s received after their child’s fifth birthday. Information on socioeconomic status (maternal education level) and cardiovascular diseases in the immediate family was also derived from this questionnaire. Maternal educational level was divided into low (primary school, technical secondary education, lower vocational secondary education), moderate (degree higher vocational secondary education, academic secondary education, intermediate vocational education) or high (degree higher vocational education, university). Cardiovascular diseases in the immediate family were answered by marking ‘no’, ‘yes’ or ‘don’t know’ for the following diseases by the mother, the child’s biological father and their first degree relatives: overweight, hypertension < 55 years, high cholesterol, stroke < 55 years, heart attack < 55 years and dead from heart attack < 55 years [25]. Information on the child’s sexual maturation at the age of 11–12 was assessed by the Tanner scale, scored by the mother [26].

.

### Health behaviours

Considered health behaviours were weight status, physical activity, diet, screen time, sleep duration and prenatal smoke exposure, apart from weight status all reported by the mother. Weight and height were measured once to calculate BMI (weight in kg/height in cm^2^). Weight was measured to the nearest 100 g by using a Marsden weighing scale (model MS-4102, Oxfordshire, UK) and height to the nearest mm with a Leicester portable height measure (Seca, Hamburg, Germany). The health check was administered at both time points by trained student assistants, trained PhD-candidates and research staff of the ABCD-study. At age 5–6 years the measurements took place at the child’s primary school except for those children enrolled in small schools, children who moved outside Amsterdam and the 11–12-year-old children. For these children, the health check took place at a central location during the weekend and holidays. For defining metabolic syndrome, waist circumference was measured placing a Seca measuring tape around the abdomen between the costal border and iliac crest. Children aged 5–6 wore underwear only, children at age 11–12 wore sport outfits. Information on physical activity included both recreational and organized physical activity outside school hours as well as transport to and from school cycling. Hours of recreational physical activity was assessed by asking the hours of playing outside at schooldays and in weekends for both summer and winter. The total hours of organized sport activities per week and the total hours of cycling to and from school were added to a total physical activity score [27].

.The FFQ that was used to evaluate the children’s food consumption consisted of questions regarding the 71 most consumed food items by Dutch children [23]. For the present study, we included information regarding fruits and vegetable intake, fish intake, fibre intake and intake of sugar-sweetened beverages in line with the healthy diet definition of the AHA [4]. The AHA guideline looks at the intake of fibre-rich whole grains, while the FFQ used in this present study measures total intake. Fibre intake was considered sufficient if it accounted 3 g/MJ, recommended by the Health council of the Netherlands [28]. Sodium intake could not be assessed with our FFQ. For other components besides fibre and sodium, the AHA cut-offs for a healthy diet were used: ≥450 g of fruits and vegetables/day, ≥2 servings of fish/week with a total weight of ≥200 g and ≤ 450 kcal of sugar-sweetened beverages/week based on a 2000 kcal diet. A comparison of our definition with the AHA definition can be found in Supplementary Table 2.

For the assessment of screen time, the number of hours per day children were watching TV, DVD or VIDEO and how many hours they played games on computer, XBOX or other types of videogames were asked. Hours of screen time for both schooldays and weekends were ranked in six categories from ‘(almost) never’ to ‘five or more hours per day’ [29]. Information on children’s sleep duration included the total hours of sleep per day, separately for schooldays and in the weekends. The average hours of sleep per day was calculated by multiplying the hours of sleep on schooldays by five and the hours of sleep in the weekend by two and then adding these two numbers together, divided by seven. For all the above stated behaviours, a weighted weekly average was calculated. Mother’s smoking habits during pregnancy were assessed from the pregnancy-questionnaire in which mothers scored the number of cigarettes smoked daily.

### Health factors

At age 5–6, plasma glucose, serum lipids and triglyceride concentrations were determined in capillary blood, collected after an overnight fast by a finger puncture by using a validated kit, developed for ambulatory purposes (Demecal: LabAnywhere, Haarlem, The Netherlands) [30]. At age 11–12, capillary blood was collected by finger puncture after 3 h fasting and analysed by the point-of-care analyser Alere Cholestech LDX machine using Lipid Profile and GLU cassettes (Cholestech Alere Health Hayward, CA, USA) [31]. At both time points, blood pressure was measured by the Omron 705 IT (Omron Health Inc., Bannockburn, IL, USA) [32] in sitting position with the arm supported at the heart level. After a test reading and 15 min of rest, blood pressure was measured twice on the upper right arm with an appropriate cuff. If these measurements differed > 10 mmHg, a third measurement was performed [33]. The systolic and diastolic blood pressure were determined by averaging the two closest measurement. The research location and research staff were similar to those reported by the calculation of weight and height.

### Defining ICH at age 5–6 years

On each ICH component, children could score ‘ideal’ (score = 1) or ‘non-ideal’ (score = 0). To define ICH components as ideal, the AHA recommendation was used for the following components: BMI <85th percentile according to the WHO growth reference [34] ideal diet when three out of four components were met [4], fasting plasma glucose < 5.6 mmol/L, total cholesterol < 4.40 mmol/L and blood pressure < 90th percentile based on reference values from the guideline of the American Academy of Pediatrics [35]. Physical activity was considered ideal according to the AHA when children were physically active at moderate or vigorous intensity for at least 60 min per day. In our study the children of mother’s who reported a total physical activity score of ≥7 h per week were considered ideal [9].

.For the extended ICH score, a score on three additional health behaviours were added. There is no evidence-based cut-off for screen time. Based on a commonly used cut-off, we considered ≤2 h of screen time per day as ideal [29]. Sleep duration was considered ideal if children slept ≥10 h per night as recommended by the National Sleep Foundation [36]. Children from mothers who reported not having smoked during pregnancy received an ideal smoking score. The definition of each component according to AHA and the additions are listed in Supplementary Table 2.

The score on extended ICH ranged from zero to nine; zero to five points was considered poor, six and seven points intermediate and eight or nine points good ICH. This subdivision was made to create roughly equal groups in size, to compare groups with relatively poor, intermediate and good cardiovascular health.

### Carotid intima-media thickness

Ultrasound measurements of the common carotid artery (CCA) were performed with the automated Panasonic Cardio Health Station V1.8 (Diagnostic Ultrasound System GM-72P00A) by three experienced ultrasound technicians at the central locations where the health check took place. During this measurement, children were in lying position with their head at a 45 degree angle. CIMT was measured bilaterally at angles 150, 120 and 90 on the right CCA and at 210, 240 and 270 on the left CCA. The software automatically identified the region of interest and froze the image. Mean CIMT was calculated by averaging the measured angles, with a minimum of at least three angles. Out of 559 individuals, 459 individuals had a CIMT measurement with at least three angles, whereof 442 individuals had all six angles measured. Increased risk CIMT was defined as a CIMT of ≥90th percentile of the cohort.

### Cardiometabolic outcomes

Secondary outcomes were glucose, total cholesterol, high-density lipoprotein cholesterol (HDL-C), triglycerides (TG), systolic and diastolic blood pressure and BMI at the age of 11–12 (all continuous). The following outcome variables were dichotomized: low HDL-C (<10th percentile), high TG (≥75th percentile), (pre) hypertension (systolic and/or diastolic blood pressure ≥ 90th percentile) and overweight (BMI ≥85th percentile). Participants were considered having metabolic syndrome when they met three or more of the following criteria: waist circumference ≥ 75th percentile, (pre) hypertension, low HDL-C, high TG and glucose ≥75th percentile [37]. All percentiles in this paragraph are based on data of the own cohort.

### Statistical analyses

We investigated the representativeness of the included subgroup by comparing the baseline characteristics between the non-included group (i.e. those who were approached at age 5–6, but did not participate in the questionnaire and/or health check at age 5–6) and the included group (i.e. those who participated both in the questionnaire and health check at age 5–6) by an independent samples t-test for continuous variables and the χ2 test for categorical variables.

#### Stage 1

Differences in demographic characteristics between the poor, intermediate and good ICH groups (based on extended ICH score) were tested by one-way analysis of variance (ANOVA) for continuous variables and the χ2 test for the categorical variables. The association of individual health behaviours, health factors and lipids with the extended ICH score at age 5–6 was evaluated by means of sex- and age adjusted multivariable linear regression models. *P*-for-trend was calculated considering ICH as a continuous variable.

#### Stage 2

We studied the association between the extended ICH score at age 5–6 with CIMT and cardiometabolic outcomes at age 11–12 by means of multivariable linear regression models for continuous outcomes and multivariable logistic regression models for dichotomous outcomes. We considered the ICH variable as a continuous variable to calculate p-for-trend. For these analyses, all models included age, sex and maturation. To examine whether the association with respect to the dichotomous outcomes was present in children with a healthy weight at baseline, we repeated the logistic regression analysis excluding children with overweight at age 5–6.

All analyses were repeated with the original ICH score.

IBM SPSS Statistic software version 20.0 (IBM Corp, Armonk, NY, USA) was used for the analysis. A *p*-value of < 0.05 was considered statistically significant.

## Results

### Comparison of included and non-included children of the ABCD-cohort

Compared to those who were not included (*N* = 2822), the included children (*N* = 1666) were younger, had more often mothers with Dutch ethnicity and a higher educational level and reported more often family history of CVD. No differences were observed in parental weight status and boy-girl distribution. (Supplementary Table 1).

#### Stage 1

##### Characteristics of the study subjects

Mean age (standard deviation (SD)) of the children at baseline was 5.7 (0.4) years. The prevalence of individual ideal ICH components were: normal glucose concentration (97.1%), no prenatal smoke exposure (92.3%), adequate physical activity (88.2%), healthy weight status (87.6%), normal blood pressure (86.9%), sufficient sleeping hours (81.4%), screen time < 2 h (80.4%), normal total cholesterol concentration (69.7%) and healthy diet (2.0%). According to the extended ICH the majority of children had either intermediate (56%) or good (33%) ICH, which means respectively 6–7 or 8–9 ideal components. Compared to children with poor ICH (11%, 1–5 ideal components), children with a good ICH were more likely to have a Western ethnicity, higher educated mothers, normal weight parents and less frequent have a family history of CVD, see Table 1.

**Table 1:** Characteristics per ICH subgroup according to the extended ICH definition

	All	Poor (1–5)	Intermediate (6–7)	Good (8–9)	*P*-value^*^
N	1666	188	934	544	
Age at first measurement (years), mean (SD)	5.7 (0.43)	5.7 (0.44)	5.7 (0.44)	5.6 (0.42)	0.055
Male gender, n (%)	846 (50.8)	101 (53.7)	471 (50.4)	274 (50.4)	0.692
Ethnicity					< 0.001
Dutch, n (%)	1210 (72.7)	88 (47.1)	677 (72.5)	445 (81.8)	
Turkey, n (%)	26 (1.6)	13 (7.0)	11 (1.2)	2 (0.4)	
Moroccan, n (%)	59 (3.5)	16 (8.6)	39 (4.2)	4 (0.7)	
Surinamese, n (%)	64 (3.8)	20 (10.7)	40 (4.3)	4 (0.7)	
Other western, n (%)	205 (12.3)	10 (5.3)	118 (12.6)	77 (14.2)	
Other non-Western, n (%)	101 (6.1)	40 (21.4)	49 (5.2)	12 (2.2)	
Maternal educational level					< 0.001
Low, n (%)	145 (8.7)	57 (31.0)	78 (8.4)	10 (1.8)	
Middle, n (%)	318 (19.2)	56 (30.4)	194 (20.8)	68 (12.5)	
High, n (%)	1195 (72.1)	71 (38.6)	659 (70.8)	465 (85.6)	
Parent weight status					< 0.001
Normal weight, n (%)	792 (48.5)	60 (33.0)	434 (47.6)	298 (55.3)	
One overweight parent, n (%)	622 (38.1)	75 (41.2)	361 (39.6)	186 (34.5)	
Two overweight parents, n (%)	218 (13.4)	47 (25.8)	116 (12.7)	55 (10.2)	
Family history of CVD					< 0.001
None, n (%)	829 (49.8)	68 (36.2)	470 (50.3)	291 (53.5)	
One parent, n (%)	590 (35.4)	79 (42.0)	322 (34.5)	189 (34.7)	
Two parents, n (%)	247 (14.8)	41 (21.8)	142 (15.2)	64 (11.8)	

*Abbreviations*: *ICH* ideal cardiovascular health, *n*,number, *SD* standard deviation, *CVD* cardiovascular diseases. ^*^Adjusted for age and sex

Table 2 shows the results of the associations between extended ICH scores and cardiovascular health outcomes at age 5–6 years. Children with higher scores had lower levels of health factors and higher percentages of ideal health behaviours.

**Table 2:** Association between extended ICH score and cardiovascular health outcomes at age 5–6 years

		Ideal Cardiovascular Health Score^**a**^								
	All	2	3	4	5	6	7	8	9	*P* for trend
N, %	1666	2 (0.1)	15 (0.9)	35 (2.1)	136 (8.2)	355 (21.3)	579 (34.8)	527 (31.6)	17 (1.0)	
Age at first measurement (years), mean (SD)	5.7 (0.4)	5.5 (0.2)	5.8 (0.5)	5.8 (0.4)	5.7 (0.4)	5.6 (0.4)	5.6 (0.4)	5.6 (0.4)	5.7 (0.5)	0.360
Male gender, n (%)	846 (50.8)	0 (0.0)	3 (20.0)	21 (60.0)	77 (56.6)	188 (53.0)	283 (48.9)	263 (49.9)	11 (64.7)	0.055
Ideal health factors at age 5/6 years										
Glucose (mmol/L), mean (SE)	4.6 (0.0)	4.6 (0.3)	4.8 (0.1)	4.9 (0.1)	4.7 (0.0)	4.6 (0.0)	4.6 (0.0)	4.5 (0.0)	4.4 (0.1)	< 0.001
Total cholesterol (mmol/L), mean (SE)	4.0 (0.0)	5.4 (0.4)	4.4 (0.2)	4.3 (0.1)	4.2 (0.1)	4.3 (0.0)	4.1 (0.0)	3.7 (0.0)	3.6 (0.2)	< 0.001
Systolic BP (mmHg) mean (SE)	97.2 (0.2)	98.4 (5.5)	105.7 (2.0)	103.7 (1.3)	101.1 (0.7)	98.4 (0.4)	96.8 (0.3)	95.3 (0.3)	92.7 (1.9)	< 0.001
Diastolic BP (mmHg), mean (SE)	57.4 (0.2)	64.3 (5.0)	66.2 (1.8)	64.2 (1.2)	60.0 (0.6)	58.5 (0.4)	57.1 (0.3)	55.5 (0.3)	56.6 (1.7)	< 0.001
HDL-C (mmol/L), mean (SE)	1.3 (0.0)	1.4 (0.2)	1.4 (0.1)	1.4 (0.0)	1.3 (0.0)	1.3 (0.0)	1.3 (0.0)	1.2 (0.0)	1.3 (0.1)	< 0.001
Triglycerides (mmol/L), mean (SE)	0.6 (0.0)	0.8 (0.2)	0.7 (0.1)	0.8 (0.0)	0.7 (0.0)	0.6 (0.0)	0.6 (0.0)	0.6 (0.0)	0.6 (0.0)	< 0.001
Ideal health behaviors 5/6y										
BMI, mean (SE)	15.4 (0.0)	18.9 (0.9)	17.9 (0.3)	17.3 (0.2)	16.1 (0.1)	15.6 (0.1)	15.3 (0.1)	15.1 (0.1)	15.3 (0.3)	< 0.001
Ideal smoking status, n (%)	1537 (92.3)	0 (0.0)	8 (53.3)	23 (65.7)	107 (78.7)	304 (85.6)	554 (95.7)	524 (99.4)	17 (100.0)	< 0.001
Ideal healthy diet score, n (%)	33 (2.0)	0 (0.0)	0 (0.0)	0 (0.0)	0 (0.0)	0 (0.0)	3 (0.5)	13 (2.5)	17 (100.0)	< 0.001
Ideal physical activity, n (%)	1469 (88.2)	1 (50.0)	7 (46.7)	21 (60.0)	90 (66.2)	286 (80.6)	521 (90.0)	526 (99.8)	17 (100.0)	< 0.001
Ideal screen time, n (%)	1339 (80.4)	0 (0.0)	4 (26.7)	8 (22.9)	49 (36.0)	228 (64.2)	506 (87.4)	527 (100.0)	17 (100.0)	< 0.001
Ideal sleep behavior, n (%)	1356 (81.4)	0 (0.0)	2 (13.3)	14 (40.0)	60 (44.1)	244 (68.7)	493 (85.1)	526 (99.8)	17 (100.0)	< 0.001

*Abbreviations*: *ICH* ideal cardiovascular health, *N* number, *SD* standard deviation, *SE* standard error, *BMI* body mass index, *BP* blood pressure, *HDL-C* high-density lipoprotein cholesterol. Results are adjusted for age and sex

^a^There were no children with 0 or 1 ICH point

#### Stage 2

##### Extended ICH score and carotid intima-media thickness

The mean CIMT at age 11–12 years was 0.462 mm (SE = 0.001). The association between extended ICH score at age 5–6 years and CIMT was not statistically significant (*p* for trend = 0.12; Table 3). Further, no statistical significant association was observed between extended ICH and the dichotomous variable ‘increased risk CIMT’ (odds ratio (OR) [95% confidence interval (95%CI)]: 1.04 [0.77–1.40], *p* = 0.80 (Table 4).

**Table 3:** Association between extended ICH score at age 5–6 years and cardiovascular health outcomes at age 11–12 years

	Ideal Cardiovascular Health Score^**a**^									
	All	2	3	4	5	6	7	8	9	P for trend
N, %	559	2 (0.4)	4 (0.7)	6 (1.1)	38 (6.8)	119 (21.3)	198 (35.4)	187 (33.5)	5 (0.9)	
Age at first measurement (years), mean (SD)	5.7 (0.4)	5.5 (0.2)	5.7 (0.4)	5.7 (0.5)	5.8 (0.5)	5.7 (0.4)	5.7 (0.5)	5.7 (0.4)	5.6 (0.5)	0.943
Age at follow up (years), mean (SD)	11.8 (0.4)	12.1 (0.3)	11.4 (0.2)	12.0 (0.4)	11.9 (0.3)	11.7 (0.4)	11.8 (0.4)	11.8 (0.3)	11.5 (0.3)	0.011
Maturation, mean (SD)	1.5 (0.5)	1.5 (0.1)	2.1 (1.1)	1.7 (0.8)	1.6 (0.5)	1.5 (0.6)	1.5 (0.6)	1.4 (0.4)	1.3 (0.5)	0.107
Cardiovascular health 11/12y										
Glucose (mmol/L), mean (SE)	4.9 (0.0)	4.5 (0.4)	4.6 (0.3)	4.8 (0.2)	5.2 (0.1)	4.9 (0.1)	4.9 (0.0)	4.9 (0.0)	4.6 (0.3)	0.920
Total cholesterol (mmol/L), mean (SE)	4.1 (0.0)	5.0 (0.4)	4.5 (0.3)	4.1 (0.3)	4.1 (0.1)	4.3 (0.1)	4.1 (0.0)	3.9 (0.0)	3.5 (0.3)	< 0.001
Systolic BP (mmHg), mean (SE)	105.2 (0.4)	110.8 (5.8)	110.6 (4.1)	113.0 (3.7)	106.8 (1.4)	105.2 (0.8)	105.1 (0.6)	104.2 (0.6)	109.3 (3.7)	0.012
Diastolic BP (mmHg), mean (SE)	60.1 (0.3)	64.4 (4.6)	63.2 (3.2)	68.9 (2.9)	60.6 (1.1)	60.5 (0.6)	60.1 (0.5)	59.1 (0.5)	66.5 (2.9)	0.011
HDL-C (mmol/L), mean (SE)	1.5 (0.0)	1.4 (0.2)	1.5 (0.2)	1.4 (0.1)	1.5 (0.1)	1.5 (0.0)	1.5 (0.0)	1.5 (0.0)	1.0 (0.2)	0.309
Triglyceriden (mmol/L), mean (SE)	1.0 (0.0)	1.0 (0.4)	0.8 (0.3)	1.2 (0.2)	1.1 (0.1)	1.0 (0.1)	0.9 (0.0)	1.0 (0.0)	0.5 (0.4)	0.468
BMI, mean (SE)	17.6 (0.1)	20.3 (1.6)	20.2 (1.1)	22.1 (1.0)	18.2 (0.4)	18.4 (0.2)	17.2 (0.2)	17.2 (0.2)	17.1 (1.0)	< 0.001
CIMT (mm), mean (SE)	0.462 (0.001)	0.445 (0.020)	0.485 (0.015)	0.429 (0.020)	0.455 (0.006)	0.458 (0.003)	0.462 (0.002)	0.464 (0.002)	0.457 (0.013)	0.123

*Abbreviations*: *N* number, *SD* standard deviation, *SE* standard error, *BP* blood pressure, *HDL-C* high-density lipoprotein cholesterol, *BMI* body mass index, *CIMT* carotid intima-media thickness

Results are adjusted for age, sex and maturation. ^**a**^There were no children with 0 or 1 ICH point.

**Table 4:** Odds ratios and 95% confidence intervals for the association between ICH score (extended and original) at age 5–6 years and cardiometabolic outcomes at age 11–12 years

	Extended definition			Original definition		
	OR^a^	95% CI	P	OR^a^	95% CI	P
(Pre)Hypertension	0.85	0.66–1.09	0.203	0.89	0.62–1.28	0.526
Metabolic Syndrome	0.81	0.66–1.01	0.056	0.89	0.64–1.22	0.456
Low HDL-C	1.05	0.78–1.41	0.757	1.30	0.84–2.02	0.232
High triglycerides	1.00	0.82–1.23	0.940	1.02	0.77–1.36	0.879
Overweight	0.54	0.43–0.68	< 0.001	0.52	0.37–0.74	< 0.001
Increased risk CIMT	1.04	0.77–1.40	0.802	1.01	0.67–1.53	0.956

*Abbreviations*: *HDL-C* high-density lipoprotein cholesterol, *CIMT* carotid intima-media thickness, *OR* odds ratio, *CI* confidence interval

^a^Adjusted for age, sex and maturation

##### Extended ICH score and cardiometabolic outcomes

The extended ICH score at age 5–6 was significantly associated with total cholesterol (*p* for trend< 0.001), systolic blood pressure (*p* for trend =0.01), diastolic blood pressure (*p* for trend = 0.01) and BMI (*p* for trend < 0.001) at age 11–12. No significant association was found for glucose (*p* for trend = 0.92), HDL-C (*p* for trend = 0.31) and triglycerides (*p* for trend = 0.47) (Table 3).

Table 4 shows OR (95%CI) for the extended ICH score and the prediction of cardiometabolic outcomes at age 11–12 years, adjusted for age-, sex- and maturation. Extended ICH was inversely associated with overweight (OR [95% CI]: 0.54 [0.43–0.68], *p* < 0.001). When children with overweight at age 5–6 were excluded, the association of ICH at age 5–6 and overweight at age 11–12 remained significant (OR: 0.66 [0.48–0.91], *p* = 0.01). No significant association was found between extended ICH score and metabolic syndrome, (pre-)hypertension, low HDL-C and high triglycerides.

### Comparison between the extended and original ICH score

Overall the results were similar regardless of using the original or extended ICH score (Supplementary Table 3). However, the prospective results revealed no statistically significant association between the original ICH score and systolic- (*p for trend* = 0.056) and diastolic blood pressure (*p for trend* = 0.212) at age 11–12 years (Supplementary Table 4). The original ICH score was inversely associated with overweight (OR [95%CI]: 0.52 [0.37–0.74, *p* < 0.001) but no longer significant when excluding children with overweight (OR [95%CI]: 0.84 [0.52–1.36], *p* = 0.47).

## Discussion

In this study, we applied an extended ICH score to a large cohort of children aged 5–6 years and investigated to what extent this score was related to cardiometabolic health at age 11–12 years. The results were compared to the original ICH score. To our knowledge this was the first time that additional components were added to the original ICH score to examine if this would improve the prediction of cardiometabolic health in children.

We found that ICH score at age 5–6 was not significantly associated with CIMT at age 11–12 according to both the extended and original ICH definition. Therefore, regarding this primary outcome, the additional criteria did not improve the prediction of CIMT, a surrogate marker for cardiovascular disease. However, both definitions showed associations of ICH score at age 5–6 with total cholesterol and BMI at age 11–12, while associations with systolic- and diastolic blood pressure were only found when using the extended definition. Both ICH-scores predicted subsequent overweight. When restricted to normal weight children at age 5–6 only, the extended ICH score still predicted overweight at age 11–12 whereas this association was no longer significant according to the original definition. In summary, the extended ICH improved prediction of some cardiovascular outcomes, but not our primary outcome.

### Interpretation of findings

#### Stage 1

Ideal health factors were more common than ideal health behaviours, but most children aged 5–6 years still scored ideal on healthy weight status (87.6%), sleeping hours (81.4%) and screen time (80.4%). Extra attention is needed for a healthy diet, since only 2% of the children met the healthy diet criteria. In line with our expectations, health factors were more often ideal than health behaviours as ideal behaviours precede changes in health factors. Other studies on ICH in child populations have shown similar findings [9–12].

.The low prevalence of a healthy diet is in line with other studies on ICH [10–12]. Another Dutch longitudinal study on the health of 8 year old Rotterdam born children found no children meeting Dutch dietary guidelines regarding intake of vegetables and sugar sweetened beverages [38]. As the Dutch dietary cut-offs were fairly similar to the AHA diet guideline [39], a similar percentage of ideal diet according to the AHA diet guideline can be expected. Dietary intake of both the Amsterdam and Rotterdam born children can thus be accounted as suboptimal.

In our study, the second most important ICH component was total cholesterol. We found 30.3% of 5–6 year old children with high total cholesterol concentrations. As lipoprotein concentrations change with growth and maturation (during puberty total cholesterol declines by 10–15%), it is questionable if the setpoint of cholesterol measurement at the age of 5–6 is representative for cardiovascular health [3, 40]. Though in studies of children > 12 years of age, the prevalence of high total cholesterol was 21.5 to 66.8% [9, 11, 12], which indicate that our findings are similar to findings of studies that examine older children. Next, of the 559 children included in stage 2 of this study, 31.7% had high total cholesterol concentrations at age 5–6, which declined to 26.8% at age 11–12.

#### Stage 2

Ideal cardiovascular health in childhood has shown to be associated with CIMT measurements in adulthood [9]. Nevertheless, clustering of CVD risk factors starts in childhood and vascular changes may therefore also occur at younger ages. In our sample ICH score at age 5–6 was not related to CIMT measurements at follow-up 6 years later. The variation of CIMT in this group was small, which can be explained by the fact that we examined a relative young and healthy population of the same age. According to the systematic review of van den Munckhof et al. [41] on CIMT in an apparently healthy adult population, 84% of CIMT can be explained by age. In contrast, several other studies in children found associations of ICH components and CIMT in childhood [14, 15, 37, 42]. The children included in these studies were mainly older at baseline (range 8 to 11) than our sample and CIMT was also measured at older ages (range 15 to 21) [14, 15, 37] However, the study by Geerts et al. [42] found an association between prenatal exposure to smoking and CIMT measurements at the age of 5 years old. The inconsistency of findings may be explained by the way in which CIMT was measured; in our study a hand guided ultrasound was used, which has a lower sensitivity than automatic devices. In addition, we performed measurements at three different angles in one segment (common carotid artery), while other studies performed measurements at one angle in three different segments (common carotid artery, internal carotid artery, carotid bulb). Lastly, different sonographers can have different findings and all studies used different readers. This inevitably makes results difficult to compare between studies.

The extended ICH definition at age 5–6 has stronger associations with systolic- and diastolic blood pressure and BMI at age 11–12 than the original definition and remained when testing only in non-overweight individuals aged 5–6 years. This difference cannot be explained by only one of the three additional components used in the extended definition. The difference is thus due to the clustering of additional components. This might be because of the bi-directional relationship between sleep duration and physical activity and sedentary behavior [43]. The implication for our study is that children with ideal levels of one of the three behaviours (sleep duration, physical activity or screen time) were more likely to have ideal levels of the other cardiovascular components, which resulted in a considerably higher ICH-level. As sleep duration, physical activity and screen time are linked, the extended definition gives a larger variance between ICH levels of those with and without ideal physical activity compared to the original ICH definition. Since there is a larger variation in ICH-level, associations of ICH-level with cardiometabolic outcomes are more likely. Furthermore, as unhealthy behaviour leads to changes in blood cholesterol concentrations which in turn leads to increased CIMT, it could be that adding more behaviours to the ICH construct will make the ICH construct more sensitive for predicting cardiovascular health.

The association between cardiometabolic outcomes and the additional components, namely: screen time, sleep behaviour and prenatal smoke exposure have been examined in previous studies [20, 21, 44]. In children, associations between sedentary behaviour and blood pressure are inconsistent [20], although there is some evidence for a relationship between short sleep duration in children and increased blood pressure [21]. Prenatal smoke exposure is not associated with an increased blood pressure in childhood according to a Portuguese study in 3–10 year old children (*n* ≈ 2500). However prenatal smoke exposure is associated with an increase in BMI (*n* = 17,286) [44]. Sleep duration in childhood is also found to be inversely associated with BMI [21], while the evidence for sedentary behaviour is inconsistent [20]. It is possible that the addition of the extra components single-handedly led to the extra association. However, it might also be that the combination of more non-ideal health behaviours is the basis of reduced cardiometabolic health which is associated with higher levels of blood pressure and BMI.

### Strengths and limitations

Our study has some important strengths, including the longitudinal study design and its large sample size. Measurements took place at the same timepoint, by the same researchers with the same measuring instruments. Further, extensive measurements were done with the validated FFQ at stage 1 in the study [24]. The present study is the first to examine ICH in childhood with CIMT measurement in children as young as 11–12 years old.

The present study also has several limitations. First, our sample differed from the ABCD-cohort in terms of ethnicity, maternal educational level and family history of CVD. As Supplementary Table 1 shows, precisely those participants are more likely to have a poor cardiovascular health. Therefore, our results probably overestimate the prevalence of ideal cardiovascular health in the ABCD-cohort. Not all children participating at age 5–6 also participated 5–6 years later, though the study cohort still consisted of 559 individuals. Information on health behaviours were reported by parents and thus all subjective. It is conceivable that parents perceive or present their child’s behaviour in a more positive manner than the actual situation is. This could lead to an overestimation of healthy behaviour, resulting in a reduction of the observed association of behaviour and disease, making it less likely that there is a significant statistical outcome [45]. Health factors were measured during the health check and automatic oscillometric devices were used for blood pressure measurements, while the blood pressure table of the American Academy of Paediatrics was made by auscultatory measurements [35]. Compared to blood pressure values measured by the auscultatory method, the oscillometric blood pressure device used in the present study found on average (SD) 4.6 (4.9) mmHg higher systolic blood pressure values and 3.3 (5.4) mmHg lower diastolic blood pressure values [32]. This could have led to an overestimation of (pre-)hypertension because of higher levels of systolic blood pressure. However, a systematically overestimation of blood pressure values would not affect the present study. Many paediatric studies now use automatic devices in combination with the standard blood pressure Tables [46], thus findings of this study are comparable with other available research. For outcome measures, our study was limited to CIMT measurement and CVD risk factors since cardiovascular events could not be studied as clinical outcome at this age.

### Implications

Our study implicates that to raise a healthy generation, there is need for dietary interventions in Dutch children. Adherence to the healthy diet guidelines among the children was extremely low, which is unfortunately since dietary habits have shown to track into adulthood [47].

As the extended definition did not improve the prediction of CIMT and most cardiometabolic factors, we do not recommend the AHA to add the extra components to their definition yet. First there is need for further research.

In compliance with other studies [48, 49], our study shows that children with a non-western ethnicity and lower educated mothers were more likely to have a poorer cardiovascular health, public health interventions regarding cardiovascular health should focus particularly on these at risk individuals.

### Recommendations for future research

Further research is needed to gain insight into the associations between lifestyle of young children and cardiovascular health later in life. We recommend other researchers to look at ICH in children under 12 years in relation to cardiometabolic outcomes at a later age and explore the addition of sleep duration, screen time and prenatal smoke exposure to ICH on these outcomes. We also encourage other researchers to examine subpopulations (i.e. overweight children). Making use of objective measures for health behaviours would be recommended.

The ABCD-cohort will also be followed up longer and report on associations of ICH at age 5–6 and cardiometabolic health at an older age.

## Conclusion

This study showed that 33% of children meet the criteria for an ideal cardiovascular health. A healthy diet was least prevalent among 5-year olds (2%). Extending the ICH score in young children with additional health behaviours improved the prediction of some cardiometabolic outcomes, but not CIMT in preadolescence, compared to the original ICH score.

## Supplementary Information

**Additional file 1: Supplementary Table 1.** Characteristics of included and non-included children. **Supplementary Table 2.** Defining Ideal Cardiovascular Health. **Supplementary Table 3.** Association between original ICH score and cardiovascular health outcomes at age 5-6 years. **Supplementary Table 4.** Association between original ICH score at age 5-6 years and cardiovascular health outcomes at age 11-12 years.

## Data Availability

The individual data are not available for a public repository for ethical reasons but can be made available to other researchers for purposes of reproducing results or for collaboration. Researchers wishing to apply for the data can contact the project leader of the ABCD-study (abcd@amsterdamumc.nl).
