# Supplementary material for: Ideal cardiovascular health at age 5–6 years and cardiometabolic outcomes in preadolescence
Source: Int J Behav Nutr Phys Act. 2021 Mar 6;18:33. doi: 10.1186/s12966-021-01090-2 (PMC7936465; doi:10.1186/s12966-021-01090-2)
Supplement: Supplementary file 1 — Additional file 1: Supplementary Table 1. Characteristics of included and non-included children. Supplementary Table 2. Defining Ideal Cardiovascular Health. Supplementary Table 3. Association between original ICH score and cardiovascular health outcomes at age 5-6 years. Supplementary Table 4. Association between original ICH score at age 5-6 years and cardiovascular health outcomes at age 11-12 years. [file 12966_2021_1090_MOESM1_ESM.docx]

**SUPPLEMENTAL MATERIAL FOR**

**Ideal cardiovascular health at age 5-6 years and cardiometabolic outcomes in preadolescence**

Hester Jaspers Faijer-Westerink, BSc^1^ Mette Stavnsbo, PhD^2,^ Barbara A. Hutten, PhD^3^, Mai Chinapaw, PhD^4^ and Tanja GM. Vrijkotte, PhD^1^

1. Department of Public and Occupational Health, Amsterdam Public Health Research Institute, Amsterdam UMC, University of Amsterdam, The Netherlands. PO Box 22700, 1100 DE Amsterdam, Meibergdreef 9, 1105 AZ Amsterdam, room J2-209 FAX:  **+ 31 20 6972316** Phone: +31 20-566 4523 Email addresses: h.h.westerink@amsterdamumc.nl, t.vrijkotte@amsterdamumc.nl
2. Department of Sports Science and Physical Education, University of Agder, Norway. PO BOX 422, 4604 Kristiansand
3. Department of Clinical Epidemiology, Biostatistics and Bioinformatics, Amsterdam Cardiovascular Sciences Research Institute, Amsterdam Public Health Research Institute, Amsterdam UMC, University of Amsterdam, Amsterdam, The Netherlands. PO Box 22700, 1100 DE Amsterdam Meibergdreef 9, 1105 AZ Amsterdam, room J1B-209-1 Email: b.a.hutten@amsterdamumc.nl
4. Department of Public and Occupational Health, Amsterdam Public Health Research Institute, Amsterdam UMC, Vrije Universiteit, Amsterdam, the Netherlands. PO Box 7057, 1007 MB Amsterdam, van der Boechorststraat 7, 1081 BT Amsterdam

*Supplementary Table 1 Characteristics of included and non-included children*

|  | Included  (n = 1,666) | Non-included  (n = 2,822) | P |
| --- | --- | --- | --- |
| Age at first measurement (years), mean (SD)  Male gender, n (%)  Ethnicity  Dutch, n (%)  Turkey, n (%)  Moroccan, n (%)  Surinamese, n (%)  Other western, n (%)  Other non-western, n (%)  Maternal educational level  Low, n (%)  Middle, n (%)  High, n (%)  Parental weight status  Normal weight, n (%)  One overweight parent, n (%)  Two overweight parents, n (%)  Family history of CVD  None, n (%)  One parent, n (%)  Two parents, n (%) | 5.7 (0.43)  846 (50.8)  1210 (72.7)  26 (1.6)  59 (3.5)  64 (3.8)  205 (12.3)  101 (6.1)  145 (8.7)  318 (19.2)  1195 (72.1)  792 (48.5)  622 (38.1)  218 (13.4)  829 (49.8)  590 (35.4)  247 (14.8) | 5.9 (0.53)  1419 (50.3)  1721 (61.0)  129 (4.6)  207 (7.3)  186 (6.6)  340 (12.1)  238 (8.4)  493 (17.8)  628 (22.6)  1653 (59.6)  1265 (46.9)  1087 (40.3)  347 (12.9)  1578 (56.1)  861 (30.6)  373 (13.3) | <0.001  0.748  <0.001  <0.001  0.370  <0.001 |

Abbreviations: n, number; SD, standard deviation; CVD, cardiovascular disease.

Supplementary Table 2 Defining Ideal Cardiovascular Health

|  | Official AHA Definition in children(1) | Original Definition used in this study | Extended definition used in this study |
| --- | --- | --- | --- |
| ***Health Factors***  Fasting plasma glucose    Total cholesterol    Blood pressure | <5.6 mmol/L (<100 mg/dL)  <4.40 mmol/L (<170 mg/dL)  <90^th^ percentile | same  same  same | same  same  same |
|  |  |  |  |
| ***Health Behaviours*** |  |  |  |
| BMI    Healthy diet              Physical activity    Screen time  Sleep behaviour  Smoking status | <85^th^ percentile  ≥450 g of fruits and vegetables/day, ≥2 servings of 100g of fish/week, ≥85gr of fibre-rich whole grains/day, <1500mg of sodium/day, and ≤450kcal of sugar-sweetened beverages/week based on a 2000kcal diet  ≥60 minutes of moderate or vigorous physical activity per day, every day  -  -  - | same  ≥450 g of fruits and vegetables/day, ≥2 servings of fish/week with a total weight of ≥200g, fibre intake of 3g/MJ and ≤450kcal of sugar-sweetened beverages/week based on a 2000kcal diet  ≥7 hours moderate or vigorous physical activity per week(2)  -  **-**  **-** | same  ≥450 g of fruits and vegetables/day, ≥2 servings of fish/week with a total weight of ≥200g, fibre intake of 3g/MJ and ≤450kcal of sugar-sweetened beverages/week based on a 2000kcal diet  ≥7 hours moderate or vigorous physical activity per week(2)  ≤2 hours of screen time per day(3)  ≥10 hours of sleep per night(4)  No smoking throughout pregnancy(5) |

Abbreviations: AHA, American Heart Association; BMI, body mass index.

Supplementary Table 3 Association between original ICH score and cardiovascular health outcomes at age 5-6 years

| N | All  1666 |  |  | **Ideal Cardiovascular Health Score^†^** |  |  |  | P for trend |
| --- | --- | --- | --- | --- | --- | --- | --- | --- |
|  |  | 1  4 (0.2%) | 2  39 (2.3%) | 3  196 (11.8%) | 4  637 (38.2%) | 5  769 (46.2%) | 6  21 (1.3%) |  |
| Age at first measurement (years), mean (SD)  Male gender, n (%)  Ideal health factors 5-6y  Glucose (mmol/L), mean (SE)  Total cholesterol (mmol/L), mean (SE)  Systolic BP (mmHg), mean (SE)  Diastolic BP (mmHg), mean (SE)  HDL-C (mmol/L), mean (SE)  Triglycerides (mmol/L), mean (SE)  Ideal health behaviours 5-6y  BMI, mean (SE)  Ideal healthy diet score, n (%)  Ideal physical activity, n (%) | 5.7 (0.43)  846 (50.8)  4.6 (0.0)  4.0 (0.0)  97.2 (0.2)  57.4 (0.2)  1.3 (0.0)  0.6 (0.0)  15.4 (0.0)  33 (2.0)  1469 (88.2) | 5.6 (0.4)  2 (50.0)  5.5 (0.2)  4.7 (0.3)  104.8 (3.8)  71.2 (3.5)  1.5 (0.1)  0.5 (0.1)  17.2 (0.6)  0 (0.0)  0 (0.0) | 5.8 (0.5)  19 (48.7)  4.9 (0.1)  4.7 (0.1)  106.3 (1.2)  64.7 (1.1)  1.4 (0.0)  0.7 (0.0)  17.3 (0.2)  0 (0.0)  18 (46.2) | 5.6 (0.4)  88 (44.9)  4.7 (0.0)  4.4 (0.0)  102.1 (0.5)  61.2 (0.5)  1.4 (0.0)  0.7 (0.0)  16.3 (0.1)  0 (0.0)  127 (64.8) | 5.7 (0.4)  316 (49.6)  4.6 (0.0)  4.3 (0.0)  97.8 (0.3)  57.7 (0.3)  1.3 (0.0)  0.7 (0.0)  15.5 (0.1)  1 (0.2)  535 (84.0) | 5.6 (0.4)  409 (53.2)  4.5 (0.0)  3.7 (0.0)  95.1 (0.3)  55.8 (0.3)  1.2 (0.0)  0.6 (0.0)  15.1 (0.0)  11 (1.4)  768 (99.9) | 5.7 (0.5)  12 (57.1)  4.4 (0.1)  3.7 (0.1)  93.0 (1.6)  56.2 (1.5)  1.3 (0.1)  0.6 (0.1)  15.2 (0.3)  21 (100.0)  21 (100.0) | 0.221  0.386  <0.001  <0.001  <0.001  <0.001  <0.001  <0.001  <0.001  <0.001  <0.001 |

*Abbreviations: ICH, ideal cardiovascular health; N, number; SD, standard deviation; SE, standard error; BMI, body mass index; BP, blood pressure; HDL-C, high-density lipoprotein cholesterol. Results are adjusted for age and sex.* **^†^***There were no children with 0 ICH point*

Supplementary Table 4 Association between original ICH score at age 5-6 years and cardiovascular health outcomes at age 11-12 years

| N | All  559 |  |  |  |  | **Ideal Cardiovascular Health Score^†^** |  | P for trend |
| --- | --- | --- | --- | --- | --- | --- | --- | --- |
|  |  | 1  1 (0.2%) | 2  8 (1.4%) | 3  68 (12.2%) | 4  226 (40.4%) | 5  251 (44.9%) | 6  5 (0.9%) |  |
| Age at first measurement (years), mean (SD)  Age at follow-up (years), mean (SD)  Maturation, mean (SD)  Cardiovascular health 11/12 years  Glucose (mmol/L), mean (SE)  Total cholesterol (mmol/L), mean (SE)  Systolic BP (mmHg), mean (SE)  Diastolic BP (mmHg), mean (SE)  HDL-C (mmol/L), mean (SE)  Triglycerides (mmol/L), mean (SE)  BMI, mean (SE)  CIMT (mm), mean (SE) | 5.7 (0.4)  11.8 (0.4)  1.5 (0.5)  4.9 (0.0)  4.1 (0.0)  105.2 (0.4)  60.1 (0.3)  1.5 (0.0)  1.0 (0.0)  17.6 (0.1)  0.462 (0.001) | 6.11  11.6  1.0  4.5 (0.5)  4.4 (0.6)  115.7 (8.2)  65.5 (6.5)  1.6 (0.3)  0.6 (0.5)  19.0 (2.3)  0.459 (0.029) | 5.9 (0.5)  11.8 (0.4)  1.7 (1.0)  4.6 (0.2)  4.8 (0.3)  110.8 (3.3)  62.2 (2.7)  1.5 (0.1)  0.8 (0.2)  19.9 (1.0)  0.461 (0.013) | 5.7 (0.4)  11.7 (0.4)  1.6 (0.6)  5.1 (0.1)  4.4 (0.1)  105.5 (1.0)  60.5 (0.8)  1.5 (0.0)  1.1 (0.1)  18.5 (0.3)  0.460 (0.004) | 5.7 (0.4)  11.8 (0.4)  1.5 (0.6)  4.9 (0.0)  4.1 (0.0)  105.5 (0.6)  60.4 (0.4)  1.5 (0.0)  0.9 (0.0)  17.7 (0.2)  0.461 (0.002) | 5.7 (0.4)  11.8 (0.3)  1.5 (0.5)  4.9 (0.0)  3.9 (0.0)  104.3 (0.5)  59.5 (0.4)  1.5 (0.0)  1.0 (0.0)  17.2 (0.2)  0.463 (0.002) | 5.6 (0.5)  11.5 (0.3)  1.3 (0.5)  4.6 (0.3)  3.5 (0.3)  109.4 (4.3)  66.6 (2.9)  1.0 (0.2)  0.5 (0.4)  17.2 (1.0)  0.457 (0.013) | 0.651  0.437  0.369  0.517  <0.001  0.056  0.212  0.073  0.572  <0.001  0.388 |

Abbreviations: N, number; SD, standard deviation; SE, standard error; BP, blood pressure; HDL, high density lipoprotein; BMI, body mass index;

CIMT, carotid intima media thickness. Results are adjusted for age, sex and maturation . **^†^**There were no children with 0 ICH point.

**SUPPLEMENTAL REFERENCES**

1. Lloyd-Jones DM, Hong Y, Labarthe D, Mozaffarian D, Appel LJ, Van Horn L, et al. Defining and setting national goals for cardiovascular health promotion and disease reduction: The american heart association’s strategic impact goal through 2020 and beyond. Circulation. 2010;121(4):586–613.

2. Laitinen T, Laitinen TT, Pahkala K, Magnussen CG, Viikari JSA, Oikonen M, et al. Ideal cardiovascular health in childhood and cardiometabolic outcomes in adulthood: The cardiovascular risk in young finns study. Circulation. 2012;125(16):1971–8.

3. Chinapaw MJM, Altenburg TM, Van Eijsden M, Gemke RJBJ, Vrijkotte TGM. Screen time and cardiometabolic function in Dutch 5-6 year olds: Cross-sectional analysis of the ABCD-study. BMC Public Health. 2014;14(1):1–6.

4. Hirshkowitz M, Whiton K, Albert SM, Alessi C, Bruni O, DonCarlos L, et al. National Sleep Foundation’s updated sleep duration recommendations: final report. SLEH [Internet]. 2015 [cited 2018 May 8];1:233–43. Available from: http://dx.doi.org/10.1016/j.sleh.2015.10.004

5. Geerts, CC, Bots, ML, Ent van der, CK, Grobbee, DE, Uiterwaal C. Parental Smoking and Vascular Damage in Their 5-year-old Children. Pediatrics [Internet]. 2012 [cited 2018 Mar 22];129(1):45–54. Available from: http://pediatrics.aappublications.org/content/pediatrics/129/1/45.full.pdf
